# Effect of type 2 diabetes on biochemical markers of bone metabolism: a meta-analysis

**DOI:** 10.3389/fphys.2024.1330171

**Published:** 2024-07-19

**Authors:** Jie Yang, Yuan Zhang, Xiaohua Liu, Binglin Chen, Le Lei

**Affiliations:** ^1^ School of Athletic Performance, Shanghai University of Sport, Shanghai, China; ^2^ Department of Rehabilitation Medicine, Shanghai Shangti Orthopaedic Hospital, Shanghai University of Sport, Shanghai, China; ^3^ The Second School of Clinical Medical College, Xuzhou Medical University, Xuzhou, Jiangsu, China

**Keywords:** type 2 diabetes, bone metabolism, bone formation, bone resorption, meta-analysis

## Abstract

**Objective:**

This meta-analysis aims to examine differences in biochemical markers of bone metabolism between individuals with type 2 diabetes (T2DM) and non-T2DM control groups.

**Materials and methods:**

Two independent evaluators searched five databases: PubMed, EMBASE, EBSCOhost, Web of Science, and the Cochrane Library. We aimed to identify observational studies investigating the impact of T2DM on biochemical markers of bone metabolism. Literature retrieval covered the period from the establishment of the databases up to November 2022. Studies were included if they assessed differences in biochemical markers of bone metabolism between T2DM patients and non-T2DM control groups using cross-sectional, cohort, or case-control study designs.

**Results:**

Fourteen studies were included in the analysis, comprising 12 cross-sectional studies and 2 cohort studies. Compared to the non-T2DM control group, T2DM patients showed reduced levels of Osteocalcin and P1NP, which are markers of bone formation. Conversely, levels of Alkaline phosphatase and Bone-specific alkaline phosphatase, other bone formation markers, increased. The bone resorption marker CTX showed decreased levels, while TRACP showed no significant difference.

**Conclusion:**

In individuals with T2DM, most bone turnover markers indicated a reduced rate of bone turnover. This reduction can lead to increased bone fragility despite higher bone mineral density, potentially increasing the risk of osteoporosis.

**Systematic Review Registration:** https://www.crd.york.ac.uk/prospero/display_record.php? identifier CRD42022366430.

## 1 Introduction

Diabetes mellitus is a common chronic condition characterized by high blood glucose levels and insulin resistance. According to the IDF diabetes map (International diabetes federation, 2021), there are about 537 million adults (ages 20–79) worldwide with diabetes, and this number is projected to increase to 643 million by 2030 and 783 million by 2045. Recent research has dientified bone as an endocrine organ that can secrete substances influencing muscle function, energy metabolism, cognitive function, and more (Komori, 2020). Type 2 diabetes mellitus (T2DM) impacts bone metabolism and raises the risk of bone-related complications, known as “Diabetic Osteopathy,” which includes conditions like osteopenia, osteoporosis, osteoarthropathy, and low-stress fractures (Wolf, 2008; Dumitru et al., 2019b; Ebrahimpur et al., 2019). Previous investigations have shown that the accumulation of advanced glycation end products (AGEs) in the body can increase the production of inflammatory cytokines and reactive oxygen species, starting a harmful cycle of chronic inflammation and bone resorption (Liu et al., 2023). This induces pro-inflammatory cytokines such as interleukin-1 beta (IL-1β), interleukin-6 (IL-6), and tumor necrosis factor-alpha (TNF-α), leading to disturbances in bone homeostasis (Sanguineti et al., 2014; Suzuki et al., 2020). Inadequate insulin levels may result in reduced bone collagen synthesis (Thrailkill et al., 2005; Tonks et al., 2017), and abnormal blood glucose levels can disrupt bone metabolism (Kanazawa, 2017). Consequently, individuals with T2DM face a higher risk of fractures. AGEs accelerate bone resorption by inhibiting osteoblast proliferation and differentiation while increasing osteoclast activity, ultimately disrupting bone metabolism and hindering bone reconstruction. However, studies have also indicated increased bone density in T2DM patients. Both bone formation by osteoblasts and bone resorption by osteoclasts are significantly influenced by energy expenditure. Elevated circulating insulin levels can increase osteoblast activity and bone formation, thereby promoting collagen synthesis. Conversely, abnormal blood glucose levels can alter blood calcium levels, stimulate parathyroid hormone secretion, and enhance bone resorption activity (Zhang et al., 2012; Eller-Vainicher et al., 2020). Thus, no definitive conclusions have been reached regarding changes in bone metabolism markers in T2DM patients.

This study aims to investigate potential variations in bone turnover indicators between T2DM patients and non-diabetic individuals, shedding light on the factors contributing to differences in bone formation and resorption markers and providing insights into the causes of osteopenia in T2DM patients.

## 2 Materials and methods

This study has been registered on the PROSPERO website under registration number CRD42022366430.

### 2.1 Inclusion and exclusion criteria

(1) Inclusion Criteria: This study is an observational investigation, including cohort studies, case-control studies, and cross-sectional studies.
(2) The study results pertain to the relationship between type 2 diabetes (T2DM) and bone metabolic indicators.
(3) Other diseases that could potentially influence bone metabolism were excluded, and the study’s cases were definitively diagnosed with T2DM. The control group consisted of healthy individuals without T2DM.
(4) The outcome measures of this study included metabolic markers of both bone formation and bone resorption.
(5) In cases where multiple articles presented data from the same study population, studies with more comprehensive data reports were selected.

Exclusion Criteria:

(1) Studies conducted on non-human populations.
(2) Review articles, experimental studies, case reports, or studies lacking control groups.
(3) Studies focusing on type 1 diabetes or other types of diabetes, as well as studies lacking clearly defined T2DM.
(4) Studies not published in English.
(5) Studies for which full-text articles or raw data were unavailable.

### 2.2 Literature retrieval strategy

A comprehensive literature search was conducted using the PubMed, Web of Science, Cochrane Library, EBSCOhost, and Embase databases. The search utilized MeSH terms such as “Diabetes Mellitus, Type 2,” “bone metabolism,” “bone turnover,” “bone formation,” “bone resorption,” and others. Two independent researchers conducted the search and obtained identical results. Any disagreements were discussed and resolved with a third reviewer. The search strategies for each database are detailed in Supplementary Material S1.

### 2.3 Literature screening and data extraction

Literature screening and data extraction were performed independently and cross-verified by two researchers. Any inconsistencies were resolved through discussion with a third reviewer. During the literature screening phase, we initially excluded duplicate publications, reviews, case reports, non-English articles, and any papers that did not meet the predefined criteria. Titles and abstracts were then carefully reviewed to eliminate clearly irrelevant studies. Finally, a thorough examination of the full text was conducted to determine study inclusion.

Data extraction included the following components: 1) Basic details of the included studies, such as title, author, year of publication, and study type. 2) Fundamental characteristics of the study subjects, including gender, age, body mass index, sample size, and disease duration. 3) Outcome indicators and corresponding measurement data. 4) Information relevant to the evaluation of study quality.

### 2.4 Evaluation of study quality

The Newcastle-Ottawa Scale (NOS) is used to evaluate the quality of cohort and case-control studies (Stang, 2010). The NOS covers three main dimensions: selection of the study population, comparability between groups, and measurement of outcomes, resulting in a total of eight items worth nine points. Studies scoring above six are categorized as high quality, those scoring six are considered medium quality, and studies scoring below six are regarded as low quality. For cross-sectional studies, the Agency for Healthcare Research and Quality (AHRQ) tool was used (Chou et al., 2018). This tool comprises 11 items, with each affirmative response scoring one point. Studies with a score blow four are classified as poor quality, while higher score indicate better quality.

### 2.5 Statistical analysis

This study was meta-analyzed using Review Manager5.3 software. Standardized Mean Difference (SMD) with 95% Confidence Intervals (Cl) was used as continuous variables. *I* ^*2*^ was used to assess the heterogeneity of the study. If *p*-value < 0.05 or *I* ^*2*^ > 50%, it was considered to be significantly heteroheneous which would use the random effect model. Otherwise, there was not heteroheneous which would use the fixed effect model. Sensitivity analysis was used to evaluate the impact of individual studies on the overall stability of the results. Finally, subgroup analyses were conducted base on various factors, including age (≥60 and <60), Body Mass Index (BMI) (≥30 and <30), duration of disease (≥10 years and <10 years), and HbA1c (≥7.5 and <7.5), to assess the impact of these factors on bone metabolism in T2DM patients.

## 3 Results

### 3.1 Retrieval process and results

**FIGURE 1:**
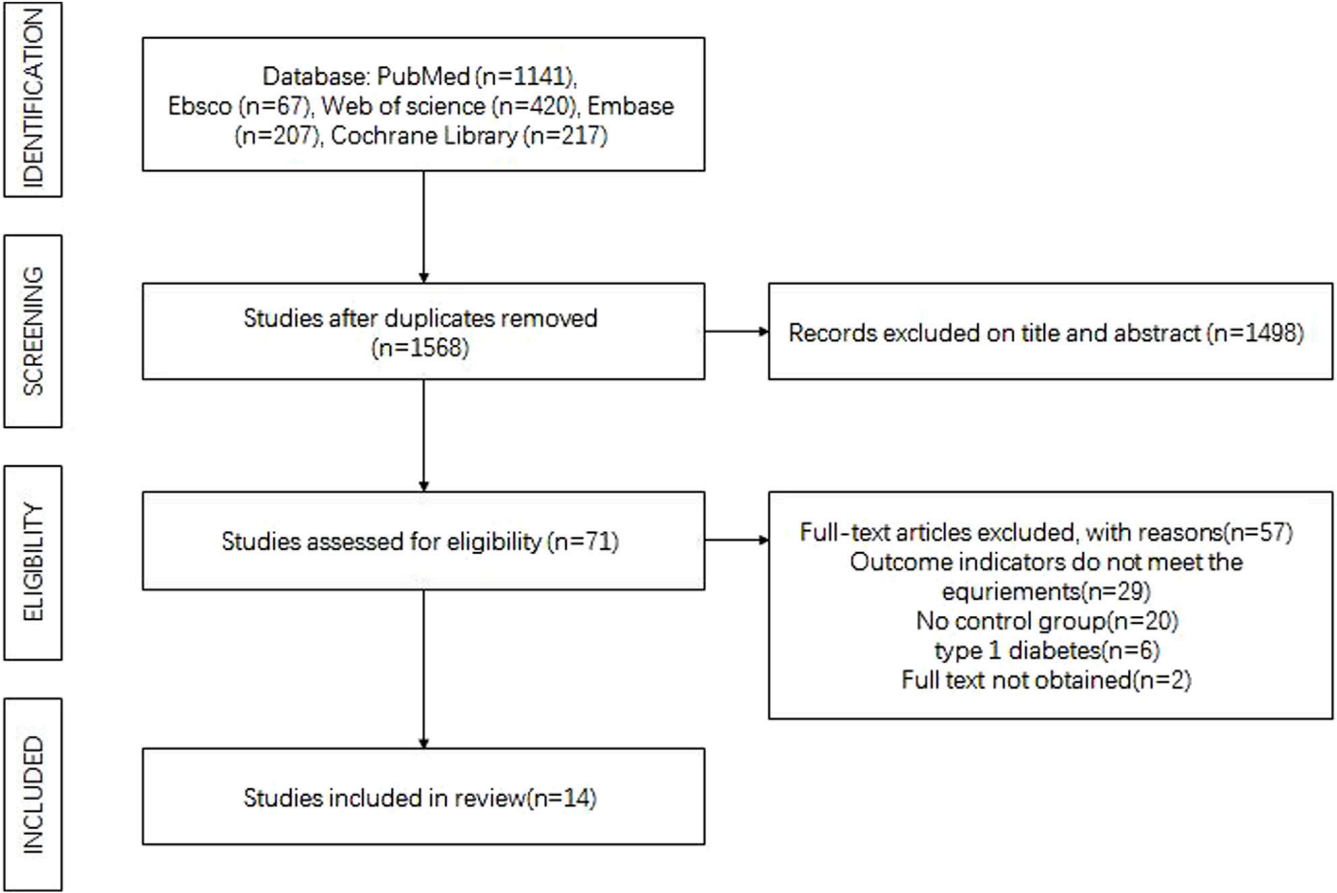
Flow chart of the literature search process.

The initial search yielded 2052 research results. After excluding 484 articles with duplicate titles and authors and another 1,498 articles unrelated to the research question based on title and abstract review, 71 studies remained for full-text assessment. Following the full-text review, 57 studies were excluded, resulting in 14 studies meeting the inclusion criteria. The details of the literature screening process and outcomes are shown in Figure 1.

### 3.2 Characteristics and quality of the included literature

This study included 14 articles (Furst et al., 2016; Wang et al., 2019; Hussein, 2017; Shu et al., 2012; Li et al., 2021; Oz et al., 2006; Akin et al., 2003; Starup-Linde et al., 2021; Purnamasari et al., 2017; Liu et al., 2020; Dumitru et al., 2019a; Reyes-García et al., 2013; Dobnig et al., 2006; Napoli et al., 2020), involving a total of 3,650 subjects: 1,680 patients with T2DM and 1970 control patients. We collected relevant information on the selected study, including the name of the first author, publication year, country, sample size, study design, patient gender, age, degree of obesity, duration of disease and HbA1c. Table 1 presents the fundamental characteristics of the subjects included in the literature.

**TABLE 1:** The basic characteristics of the research objects.

References	Country	Study design	Sample size		Sex (M/F)		Age (y)		BMI (kg/m2)		Duration of T2DM(years)	HbA1c(%)	
			CTR	T2DM	CTR	T2DM	CTR	T2DM	CTR	T2DM		CTR	T2DM
Furst et al. (2016)	USA	CSS	19	16	0/19	0/16	65.60 ± 1.20	65.40 ± 2.40	30.50 ± 1.30	31.50 ± 1.60	14.30 ± 2.00	5.80 ± 0.40	8.30 ± 1.60
Wang et al. (2019)	China	CSS	93	237	0/93	0/237	64.61 ± 7.62	64.41 ± 9.23	Not mentioned	12.08 ± 8.64	12.08 ± 8.64	5.67 ± 0.33	9.36 ± 2.35
Hussein (2017)	Egypt	CSS	20	20	13/7	11/9	49.80 ± 8.50	57.80 ± 5.90	Not mentioned	Not mentioned	11.40 ± 6.20	Not mentioned	Not mentioned
Shu et al. (2012)	USA	CSS	25	25	Not mentioned	Not mentioned	60.40 ± 14.00	63.40 ± 7.00	29.20 ± 5.00	30.60 ± 6.00	8.50 ± 7.00	Not mentioned	7.90 ± 2.00
Li et al. (2021)	China	CSS	152	88	0/152	0/88	65.82 ± 10.71	63.55±8.99	23.21 ± 3.97	24.71 ± 3.77	Not mentioned	5.75 ± 0.35	10.98 ± 2.48
Oz et al. (2006)	Turkey	CSS	48	52	14/34	15/37	52.23 ± 6.04	54.00 ± 6.34	28.53 ± 4.67/29.82 ± 5.58 (M/F)	26.74 ± 3.51/31.70 ± 4.19 (M/F)	4.80 ± 4.90	Not mentioned	Not mentioned
Akin et al. (2003)		CSS	20	57	0/20	0/57	54.35 ± 3.17	53.2 ± 4.16	26.73 ± 4.07	29.69 ± 3.72	6.10 ± 5.31	5.10 ± 0.49	9.76 ± 2.38
Starup-Linde et al. (2021)	Denmark	CSS	98	98	50/48	50/48	58.00 ± 9.70	58.00 ± 9.70	26.00 ± 4.00	30.00 ± 4.70	2.00 ± 1.40	5.60 ± 0.30	6.50 ± 0.60
Purnamasari et al. (2017)	Indonesia	CSS	40	41	0/40	0/41	40.42 ± 6.15	45.00 ± 6.15	25.71 ± 4.61	26.71 ± 6.15	8.65 ± 5.38	5.50 ± 0.46	9.75 ± 3.00
Liu et al. (2020)	China	CSS	69	160	69/0	160/0	41.80±5.10	41.00 ± 5.90	27.27 ± 3.68	27.89 ± 4.28	4.70 ± 6.00	Not mentioned	8.69 ± 1.84
Dumitru et al. (2019a)	Romania	CSS	83	56	0/83	0/56	60.21 ± 8.77	63.57 ± 8.97	27.59 ± 4.73	31.04 ± 4.55	Not mentioned	5.47 ± 0.31	6.84 ± 1.24
Reyes-García et al. (2013)	Spain	CSS	55	78	Not mentioned	Not mentioned	53.27 ± 20.55	52.3 ± 22.66	29.74 ± 12.18	34.94 ± 27.95	13.30 ± 7.60	Not mentioned	8.01 ± 1.90
Dobnig et al. (2006)	Austria	CS	1081	583	Not mentioned	Not mentioned	84.20 ± 6.30	82.80 ± 5.90	25.40 ± 4.80	26.40 ± 4.60	Not mentioned	5.50 ± 0.20(nonfasting)	6.50 ± 0.90(nonfasting)
Napoli et al. (2020)	United States	Cohort	167	169	83/84	92/77	73.50 ± 2.80	73.30 ± 2.80	26.70±4.40	29.10 ± 4.90		Not mentioned	Not mentioned

M, male; F, female; CS, cohort study; CSS, cross-sectional study.

Among the 14 observational studies analyzed, 12 were cross-sectional studies, and 2 were cohort studies. The AHRQ scores for the 12 cross-sectional studies ranged from seven to nine points, with one scoring six points, indicating a high level of quality. According to the NOS criteria, the 2 cohort studies scored six and eight, respectively. All 14 included studies achieved NOS and AHRQ scores of six or higher, signifying high study quality. Additional details regarding study quality scores can be found in Table 2.

**TABLE 2:** Aspects of quality and design of the included articles.

References	scale	Define the source of information (survey, record review)	List inclusion and exclusion criteria for exposed and unexposed subjects (cases and controls) or refer to previous publications	Indicate time period used for identifying patients	Indicate whether or not subjects were consecutive if not population-based	Indicate if evaluators of subjective components of study were masked to other aspects of the status of the participants	Describe any assessments undertaken for quality assurance purposes (e.g., test/retest of primary outcome measurements)	Explain any patient exclusions from analysis	Describe how confounding was assessed and/or controlled.	If applicable, explain how missing data were handled in the analysis	Summarize patient response rates and completeness of data collection	Clarify what follow-up, if any, was expected and the percentage of patients for which incomplete data or follow-up was obtained	score
Furst et al. (2016)	AHRQ	Y	Y	Y	Y	Y	Y	Y	Y	N	Y	N	9
Wang et al. (2019)	AHRQ	Y	Y	Y	Y	Y	Y	Y	Y	N	Y	N	9
Hussein (2017)	AHRQ	Y	Y	N	N	Y	Y	N	Y	N	Y	N	6
Shu et al. (2012)	AHRQ	Y	Y	N	Y	Y	Y	N	Y	Y	Y	N	8
Li et al. (2021)	AHRQ	Y	Y	Y	Y	Y	Y	Y	Y	N	Y	N	9
Oz et al. (2006)	AHRQ	Y	N	N	Y	Y	Y	Y	Y	Y	Y	N	8
Akin et al. (2003)	AHRQ	Y	Y	Y	N	Y	Y	N	Y	N	Y	N	7
Starup-Linde et al. (2021)	AHRQ	Y	Y	N	N	Y	Y	Y	Y	Y	Y	N	8
Purnamasari et al. (2017)	AHRQ	Y	Y	Y	Y	Y	Y	Y	Y	N	Y	N	9
Liu et al. (2020)	AHRQ	Y	Y	Y	N	Y	Y	N	Y	N	Y	N	7
Dumitru et al. (2019a)	AHRQ	Y	N	Y	Y	Y	Y	N	Y	N	Y	N	7
Reyes-García et al. (2013)	AHRQ	Y	Y	Y	Y	Y	Y	N	Y	N	Y	N	8

		Representativeness of the exposed cohort	Selection of the non exposed cohort	Ascertainment of exposure	Demonstration that outcome of interest was not present at start of study	select the most important factor	study controls for any additional factor	Assessment of outcome	Was follow-up long enough for outcomes to occur	Adequacy of follow up of cohorts			
Dobnig et al. (2006)	HOS	Y	Y	Y	N	N	N	Y	Y	Y			6
Napoli et al. (2020)	HOS	Y	Y	Y	N	Y	Y	Y	Y	Y			8

AHRQ, American Agency for Healthcare Research and Quality; NOS, Newcastle-Ottawa Scale. Y, Yes; N, No.

### 3.3 Meta-analysis results

#### 3.3.1 Differences in bone formation metabolic markers between T2DM patients and the control group

**FIGURE 2:**
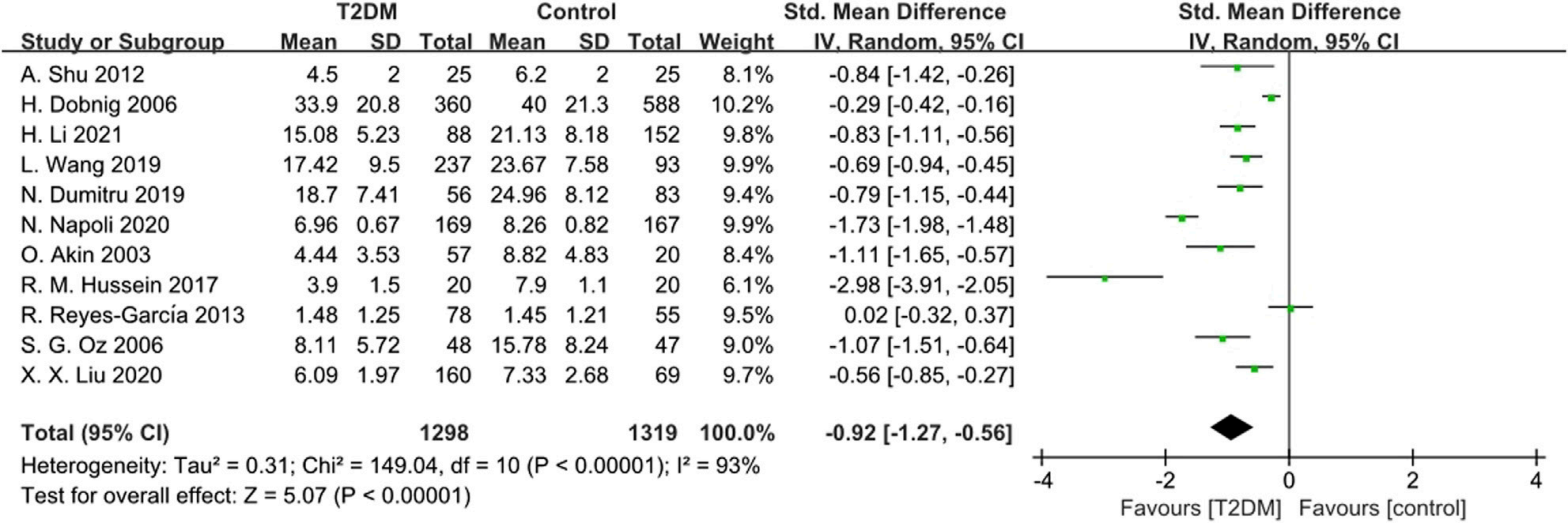
Forest plot for comparison of Osteocalcin (OC) (ng/mL) between T2DM patients and control group.

Eleven studies provided data on OC. Due to significant heterogeneity (*p* < 0.00001, *I* ^*2*^ = 93%), we used a random-effects model to analyze the Standardized Mean Difference (SMD) of OC. The findings showed that the OC levels in the T2DM group were lower compared to the healthy control group (SMD = −0.92, 95% CI = −1.27 to −0.56, *p* < 0.00001, Figure 2), and this difference was statistically significant. Sensitivity analysis was performed by systematically excluding each study, confirming the stability of the meta-analysis outcome.

**FIGURE 3:**
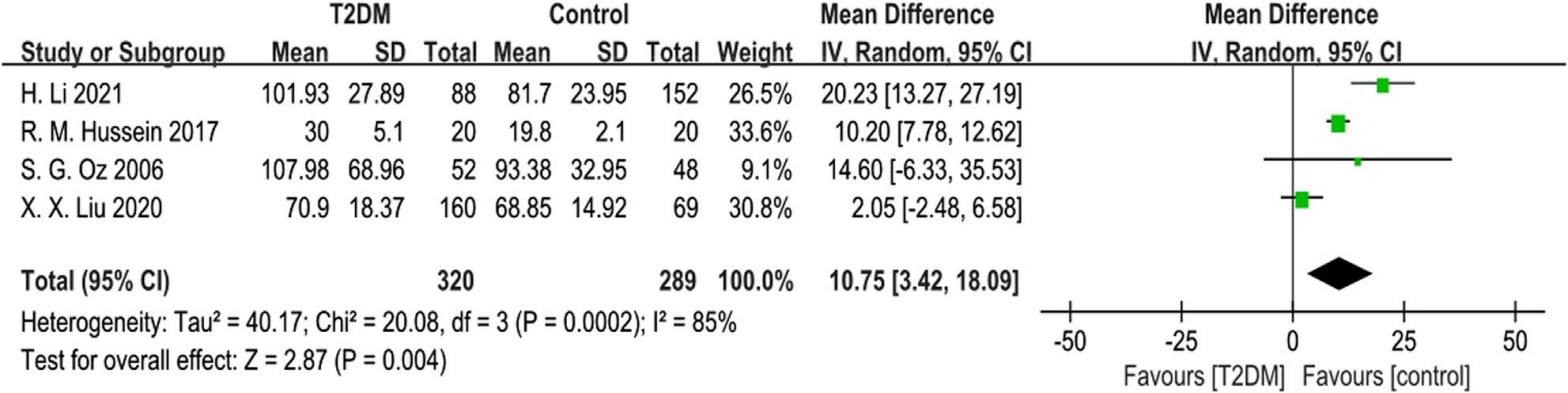
Forest plot for comparison of Alkaline phosphatase (ALP) (μg/L) between T2DM patients and control group.

Four studies reported ALP data. Given substantial heterogeneity (*p* = 0.0002, *I* ^*2*^ = 85%), we used a random-effects model to combine the ALP data. The results showed that ALP levels in the T2DM group were higher than those in the control group (SMD = 10.75, 95% CI = 3.42 to 18.09, *p* = 0.004, Figure 3), and this difference was statistically significant. Sensitivity analysis, conducted by sequentially excluding each study, confirmed the stability of the meta-analysis results.

**FIGURE 4:**
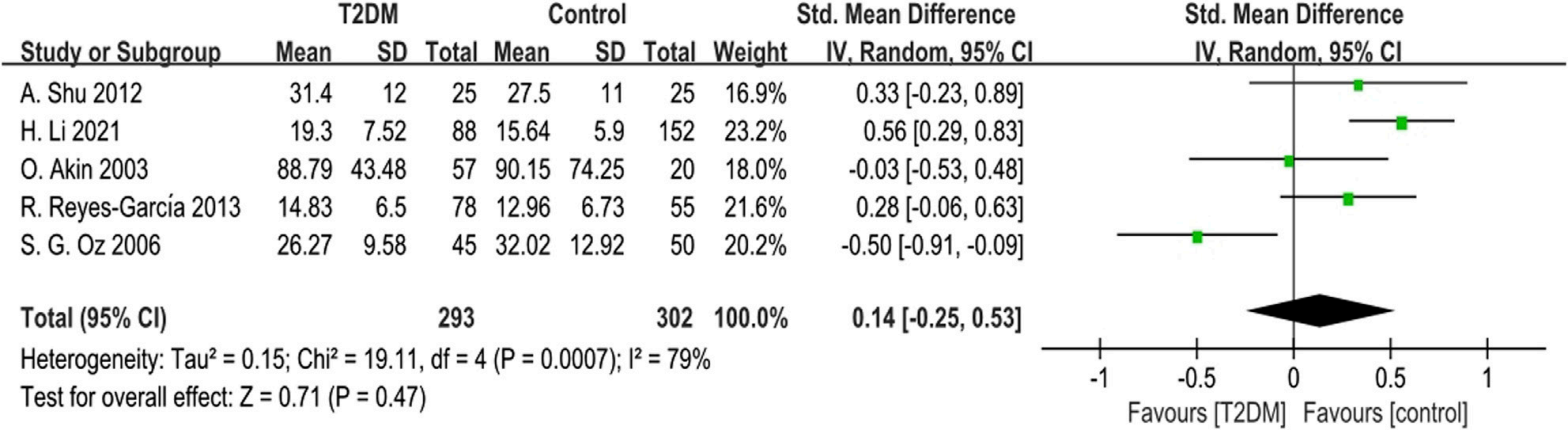
Forest plot for comparison of Bone-specific alkaline phosphatase (BSAP) (μg/L) between T2DM patients and control group.

Five studies provided BSAP data. Due to notable heterogeneity (*p* = 0.0007, *I* ^*2*^ = 79%), we used a random-effects model to combine the BSAP data. The findings indicated no statistically significant difference in BSAP levels between the T2DM group and the control group (SMD = 0.14, 95% CI = −0.25 to 0.53, *p* = 0.047, Figure 4). Sensitivity analysis, involving the sequential exclusion of each study, showed significant differences in results after removing the S G Oz (2006), suggesting some instability in the meta-analysis results.

**FIGURE 5:**
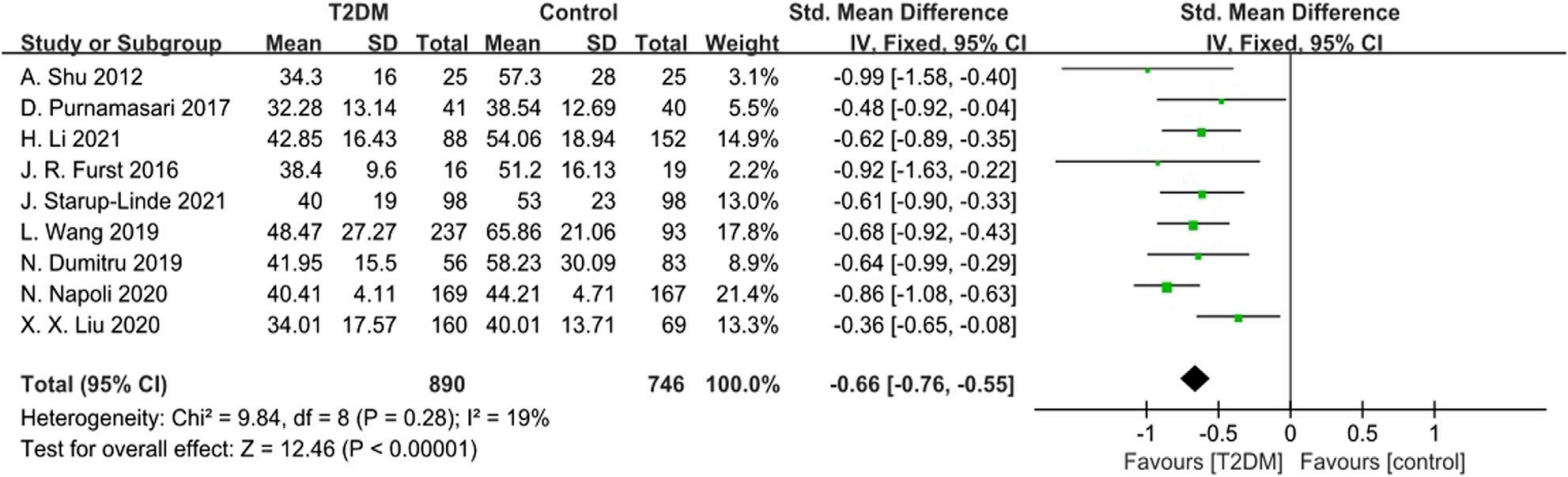
Forest plot for comparison of N-terminal propeptide of type I procollagen (P1NP) (ng/mL) between T2DM patients and control group.

Nine studies provided data on P1NP. The heterogeneity test results showed low heterogeneity (*p* = 0.28, *I* ^*2*^ = 19%) among these studies. We used a fixed-effects model to analyze the combined SMD of P1NP. The outcomes showed that P1NP levels in the T2DM group were lower than those in the control group (SMD = −0.66, 95% CI = −0.76 to −0.55, *p* < 0.00001, Figure 5), and this difference was statistically significant. Sensitivity analysis, involving the sequential exclusion of each study, confirmed the stability of the meta-analysis results.

One study reported data on C-terminal propeptide of type I procollagen (T1CP) (ng/mL), showing no statistically significant difference in T1CP levels between the T2DM group and the control group.

#### 3.3.2 The metabolic markers of bone resorption were different between T2DM patients and control group

**FIGURE 6:**
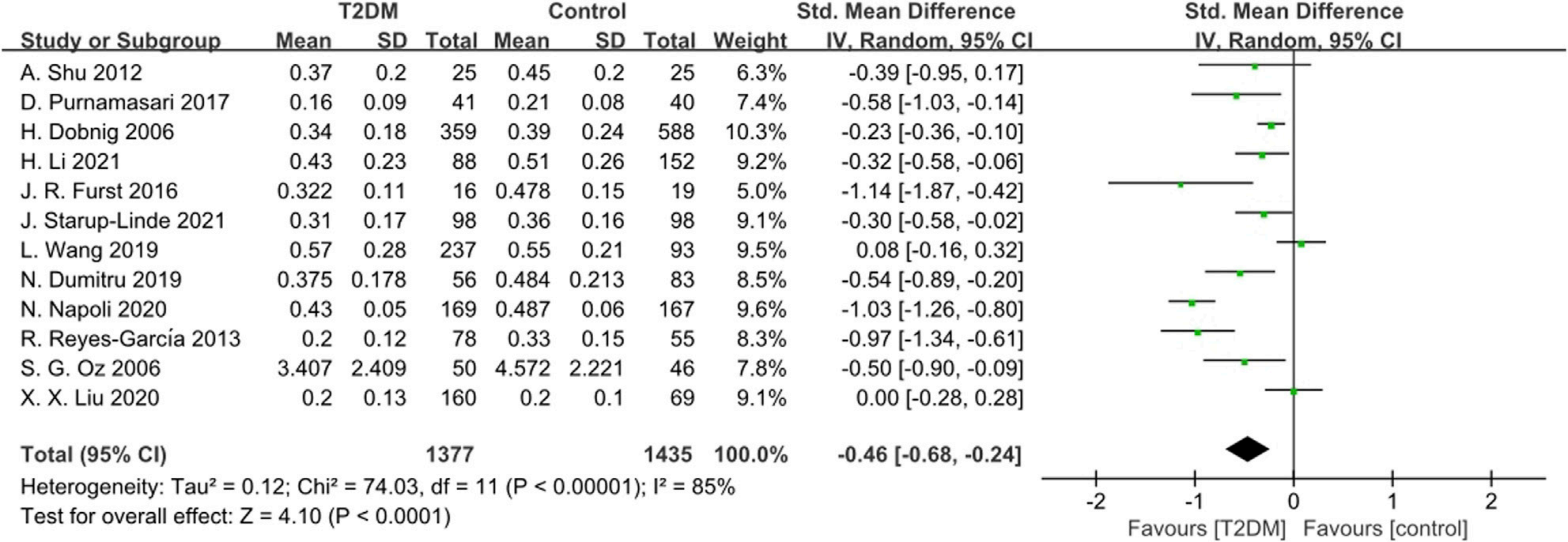
Forest plot for comparison of C-telopeptide of type 1 collagen (CTX) (ng/mL) between T2DM patients and control group.

Twelve studies reported CTX data. The heterogeneity test revealed significant heterogeneity (*p* < 0.0001, *I* ^*2*^ = 85%) among these studies. Therefore, we used a random-effects model to analyze the combined SMD of CTX. The results showed that CTX levels in the T2DM group were lower than those in the control group (SMD = −0.46, 95% CI = −0.68 to −0.24, *p* < 0.0001, Figure 6), and this difference was statistically significant. Sensitivity analysis, conducted by systematically excluding each study, confirmed the stability of the meta-analysis results.

**FIGURE 7:**
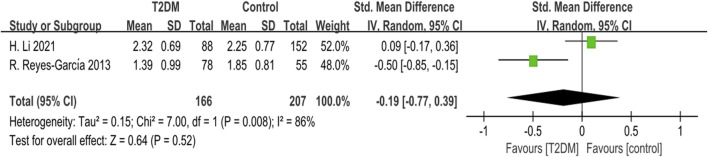
Forest plot for comparison of Tartrate-resistant acid phosphatase (TRACP) (U/L) between T2DM patients and control group.

Two studies reported TRACP data. The heterogeneity test indicated significant heterogeneity (*p* = 0.008, *I* ^*2*^ = 86%) among these studies. Using a random-effects model, we found no statistically significant difference in TRACP levels between the T2DM group and the control group (SMD = −0.19, 95% CI = −0.77 to 0.39, *p* = 0.52, Figure 7).

One study reported data on N-terminal peptides of type I collagen/creatinine (NTX/Cr), revealed a statistically significant difference in NTX/Cr between the T2DM group and the control group (*p* < 0.001). In contrast, another study reported serum NTX (nmol/BCE/L) data, but the results indicated no statistically significant difference (*p* = 0.1) between the T2DM group and the control group.

Additionally, one study provided data on deoxypyridinoline (DPD) (nM DPD/mM Cr), showing no statistically significant difference in DPD levels between the T2DM group and the control group.

#### 3.3.3 Subgroup analysis

Subgroup analysis was performed for OC, P1NP and CTX. The effects of age (≥60 and <60), body mass index (≥30 and <30), duration of disease (≥10 years and <10 years) and HbA1c (≥7.5% and <7.5%) on bone metabolic markers in T2DM patients were examined. It was found that there was no significant difference in OC results between obese T2DM patients and the control group, while there was a significant difference between non-obese T2DM patients and the control group. There was no significant difference between T2DM patients with disease duration of more than 10 years and the control group, while there was a significant difference between T2DM patients with disease duration of less than 10 years. There was no significant difference in age or HbA1c control level between T2DM patients and non-T2DM patients. CTX results were not significantly different between patients with type 2 diabetes and controls with different levels of obesity, but the I^2^ was higher (I^2^ = 86%), and the results should be treated with caution. The analysis results are shown in Table 3.

**TABLE 3:** Summary of subgroup analysis results.

Bone metabolism markers	subgroup			No of T2DM	No of control	Mean difference(95% CI)	Heterogeneity(I²%)	Effect size P
	factor	group	No.of study					
OC	Age	≥60	6	935	1108	−4.41 [−6.48, −2.34]	94	<0.0001
		60	5	363	211	−3.13 [−5.17, −1.08]	96	0.003
	BMI	≥30	3	159	163	−2.26 [−4.72, 0.19]	93	0.07
		30	6	1071	1089	−3.91 [−5.48, −2.34]	93	<0.00001
	Duration	≥10	3	335	168	−3.31 [−6.85, 0.22]	98	0.07
		10	4	290	161	−3.29 [−5.28, −1.30]	87	0.001
	HbA1c	≥7.5	6	645	414	−0.64 [−0.92, −0.36]	75	<0.00001
		7.5	2	416	671	−0.51 [−1.01, −0.02]	86	0.04
P1NP	Age	≥60	6	591	539	−13.24 [−19.84, −6.65]	90	<0.0001
		60	3	299	207	−8.12 [−12.35, −3.89]	50	0.0002
	BMI	≥30	4	195	225	−14.77 [−18.68, −10.86]	0	<0.00001
		30	5	695	521	−8.61 [−13.32, −3.91]	88	0.0003
	Duration	≥10	2	253	112	−16.06 [−20.71, −11.42]	0	<0.00001
		10	4	324	232	−10.15 [−15.64, −4.65]	68	0.0003
	HbA1c	≥7.5	6	567	398	−11.57 [−16.09, −7.06]	70	<0.00001
		7.5	2	154	181	−14.23 [−18.90, −9.55]	0	<0.00001
CTX	Age	≥60	7	950	1127	−0.06 [−0.09, −0.03]	62	<0.00001
		60	5	427	308	−0.06 [−0.12, −0.00]	85	0.04
	BMI	≥30	5	273	280	−0.10 [−0.14, −0.06]	49	<0.00001
		30	6	1054	1109	−0.04 [−0.06, −0.01]	72	0.002
	Duration	≥10	3	331	167	−0.09 [−0.20, 0.02]	90	0.12
		10	5	374	278	−0.04 [−0.08, 0.00]	67	0.08
	HbA1c	≥7.5	7	645	453	−0.06 [−0.11, −0.02]	82	0.009
		7.5	3	513	769	−0.06 [−0.09, −0.03]	30	<0.0001

## 4 Discussion

Bone turnover biomarkers (BTMs) serve as indicators of bone metabolism, divided into bone formation and bone resorption, markers, reflecting the activities of osteoblasts and osteoclasts, respectively. Notable markers of bone formation include OC, ALP, P1NP, and P1CP of bone resorption include CTX, NTX, TRACP, and DPD (Greenblatt et al., 2017). Serum OC is released by osteoblasts during the bone formation PINP and PICP are markers for the secretion of type 1 collagen by osteoblasts, with P1NP being a degradation product formed during type I collagen synthesis. Thus, P1NP and OC are crucial markers of bone formation. β-CTX is a product of the breakdown of mature type I collagen by osteoclasts, making it a key marker of bone resorption. CTX and NTX are fragments of type I collagen from the telopeptide region cleaved during osteoclast activity, released into the circulation at a rate proportional to bone resorption. Therefore, β-CTX is a vital marker of bone resorption. DPD, primarily found in bone, is considered a specific marker for bone turnover.

In this study, the bone formation markers OC and P1NP generally showed lower levels in T2DM patients compared to the control group, whereas ALP and BSAP showed higher levels in T2DM patients. Conversely, P1CP did not show a significant difference between T2DM patients and the control group. These findings align with those of Hygum K (Hygum et al., 2017), who reported reduced levels of bone turnover markers in both diabetic and non-diabetic control groups. It is important to note that T2DM typically manifests at a later age than type 1 diabetes, and their underlying causes differ. Therefore, the reasons for the reduced levels of bone turnover markers may vary. OC can be categorized into carboxylated and non-carboxylated forms, with carboxylated OC primarily associated with the bone matrix and uncarboxylated OC (ucOC) present in the circulatory system. ucOC plays a crucial role in mineral balance within bones, binding to calcium and regulating glucose levels in the body (Alamri et al., 2023). Serum OC levels are closely linked to the quantity and activity of osteoblasts, as well as the rates of both new bone formation and old bone resorption (Patsch et al., 2013). The inhibition of bone formation could be due to the accumulation of advanced glycation end products within the organic bone matrix, potentially disrupting normal osteoblast functionality. Experimental evidence from animal and *in vitro* studies suggests that hyperglycemic environments can suppress osteoblast activity, thereby decreasing OC and P1NP levels. Nearly 50% of circulating alkaline phosphatase originates from bone tissue. Results concerning ALP and BSAP have varied among different studies. Some studies have observed increased BSAP expression in conditions of chronic hyperglycemia, possibly due to elevated levels of reactive oxygen species (ROS) induced by high blood glucose (Botolin and McCabe, 2006). BSAP produced by osteoblasts during bone formation serves to deactivate pyrophosphate, an inhibitor of mineralization. Elevated BSAP levels can enhance bone mineralization, potentially explaining the increased bone density observed in T2DM patients. P1NP, synthesized from specific procollagen precursors, serves as a preferred indicator of bone formation. Increased glucose exposure in T2DM can lead to the accumulation of AGEs which may disrupt osteoblast differentiation and reduce osteoblast levels (Kume et al., 2005). Prolonged hyperglycemia can impair osteoblast maturation, overall bone formation, and mineralization. The combination of low bone formation and high mineralization may indicate increased bone mineralization in T2DM patients, potentially explainning the paradox of higher bone density coupled with reduced bone strength (Starup-linde and Vestergaard, 2016).

Regarding bone resorption markers, CTX levels were lower in T2DM patients compared to the control group, while TRACP and DPD showed no statistically significant differences. NTX was mentioned in only two studies, with inconclusive results were. β-CTX released into the bloodstream during the degradation of type I collagen by osteoclasts, serves as a sensitive marker of osteoclastic activity and is internationally recognized as an indicator of bone resorption. In animal models, increased bone resorption levels were associated with elevated serum levels of CTX, TRACP, and histological evidence of osteoclast activity (Eckhardt et al., 2020). However, in clinical settings, variations in disease duration, metabolic status, and genetic backgrounds among patients have led to ongoing debates regarding changes in bone resorption markers. Research has indicated that metformin, a common diabetes medication, can influence bone metabolic markers. Blood glucose regulation also plays a critical role in bone metabolism. It is worth noting that DPD is primarily measured in urine, and its results are closely linked to renal function. The reliability of this marker as an indicator of bone resorption requires further validation.

Significant heterogeneity (*I* ^*2*^ > 72%) was observed among the bone formation biomarkers OC, ALP, and the bone resorption biomarker CTX. However, excluding individual studies did not substantially alter the heterogeneity, indicating the stability of the research results. Notably, removing of the Oz’s study (Oz et al., 2006) decreased *I* ^*2*^ from 79% to 33% for the bone formation marker BSAP, suggesting relatively low result stability for BSAP. Therefore, caution should be exercised when interpreting differences in BSAP levels between T2DM patients and healthy individuals, considering its role as a bone formation biomarker.

In the subgroup analysis, we found no significant difference in OC results between obese T2DM patients and controls, but significant differences between non-obese T2DM patients and controls, indicating that obesity may play a role in promoting OC activity in T2DM patients, and OC activity is decreased in T2DM patients. Some researchers believe that although OC regulates bone formation, it is also regulated by other hormones (such as insulin), affecting adipose tissue (Ramirez Ruiz et al., 2023). Insulin resistance reduces the binding of insulin to osteoblasts, and OC secretion is insufficient. Allamri has studied the association between OC and BMI in T2DM patients in Saudi Arabia (Alamri et al., 2024), and he also found that OC was higher in the obese group than in the non-obese T2DM patients group, which was consistent with the conclusion of this study. Duration of T2DM more than 10 years of OC there was no significant difference with control group, and duration less than 10 years of T2DM patients have significant difference. A study on a survey of Denmark in T2DM patients and non-T2DM patients in changes in the rate of fractures (Kvist et al., 2023), found in T2DM patients have a higher rate of fracture, and fracture rate over time, so the duration of disease is also a possible influencing factor for OC indicators in T2DM patients. The index of bone resorption CTX did not change significantly in different periods of T2DM patients and did not differ significantly from the control group. Although some studies have shown that HbA1c is an important indicator affecting bone metabolism (Meier et al., 2023), there were significant differences between well-controlled and poorly controlled T2DM patients and controls in both OC and CTX, and there was no significant difference in bone turnover in T2DM patients with glucose control. Markers of bone turnover are generally sensitive. Subjects’ race, measurement method, drug use, complications, *etc.*, all have an impact on the results, which may be the reason for the large heterogeneity of the results. Due to the incomplete data collected by the included articles, the results of subgroup analysis need to be further compensated by more clinical studies of higher quality.

Several limitations are inherent in our study. The inclusion of observational studies introduces some degree of selection bias, due to study design, making them inherently less precise and accurate compared to randomized controlled trials. Despite the importance of confounders such as fasting glucose levels, diabetes duration, BMI, and pharmacological interventions, the absence of stratified data on bone metabolism in the included reports limits our ability to further explore bone turnover indicators in T2DM further.

## 5 Conclusion

Our meta-analysis revealed that bone turnover rates were lower in T2DM patients compared to non-diabetic individuals. Specifically, bone formation markers OC and P1NP exhibited lower levels in T2DM patients, while ALP and BSAP levels were higher in this group compared to non-diabetic individuals. T2DM patients with a disease duration of less than 10 years or who are not obese can consider OC as a predictor of fracture risk. Additionally, T2DM patients showed lower levels of the bone resorption marker CTX compared to those without diabetes. The decrease in most bone turnover markers among T2DM patients suggests a reduced bone turnover rate, contributing to increased bone fragility despite higher bone mineral density. This heightened risk of osteoporosis underscores the importance of bone turnover markers as valuable indicators for assessing fracture risk in individuals with T2DM.

## Data Availability

The original contributions presented in the study are included in the article/Supplementary Material, further inquiries can be directed to the corresponding authors.
