# Supplementary material for: Effect of type 2 diabetes on biochemical markers of bone metabolism: a meta-analysis
Source: Front Physiol. 2024 Jul 19;15:1330171. doi: 10.3389/fphys.2024.1330171 (PMC11294215; doi:10.3389/fphys.2024.1330171)
Supplement: Supplementary file 1 [file Table1.DOCX]

Pubmed:

**#1**

**((((((Diabetes mellitus, type 2[Title/Abstract]) OR (Type 2 diabetes mellitus[Title/Abstract])) OR (Type 2 diabetes[Title/Abstract])) OR (Type 2 diabetic[Title/Abstract])) OR (T2DM[Title/Abstract])) OR (T2D[Title/Abstract])) OR ("Diabetes Mellitus, Type 2"[Mesh])**

**222185**

**#2**

**(((((((((((((((bone remodeling[MeSH Terms]) OR (osteogenesis[MeSH Terms])) OR (osteocalcin[MeSH Terms])) OR (osteoprotegerin[MeSH Terms])) OR (alkaline phosphatase[MeSH Terms])) OR (procollagen type I N-terminal peptide[MeSH Terms])) OR (ossification[Title/Abstract])) OR (bone metabolism[Title/Abstract])) OR (bone formation[Title/Abstract])) OR (osteogenesis[Title/Abstract])) OR (bone turnover[Title/Abstract])) OR (bone remodeling[Title/Abstract])) OR (osteocalcin[Title/Abstract])) OR (osteoprotegerin[Title/Abstract])) OR (alkaline phosphatase[Title/Abstract])) OR (procollagen type I N-terminal peptide[Title/Abstract])**

**256480**

**#3**

**((((((((bone resorption[MeSH Terms]) OR (collagen type I trimeric cross-linked peptide[MeSH Terms])) OR (tartrate resistant acid phosphatase[MeSH Terms])) OR (bone resorption[Title/Abstract])) OR (collagen type I trimeric cross-linked peptide[Title/Abstract])) OR (C-terminal telopeptide[Title/Abstract])) OR (N-terminal telopeptide[Title/Abstract])) OR (deoxypyridinoline[Title/Abstract])) OR (tartrate resistant acid phosphatase[Title/Abstract])**

**64168**

#4 274842

#5 1911

#6

#7

**((((((((Diabetes mellitus, type 2[Title/Abstract]) OR (Type 2 diabetes mellitus[Title/Abstract])) OR (Type 2 diabetes[Title/Abstract])) OR (Type 2 diabetic[Title/Abstract])) OR (T2DM[Title/Abstract])) OR (T2D[Title/Abstract])) OR ("Diabetes Mellitus, Type 2"[Mesh])) AND (((((((((((((((((bone remodeling[MeSH Terms]) OR (osteogenesis[MeSH Terms])) OR (osteocalcin[MeSH Terms])) OR (osteoprotegerin[MeSH Terms])) OR (alkaline phosphatase[MeSH Terms])) OR (procollagen type I N-terminal peptide[MeSH Terms])) OR (ossification[Title/Abstract])) OR (bone metabolism[Title/Abstract])) OR (bone formation[Title/Abstract])) OR (osteogenesis[Title/Abstract])) OR (bone turnover[Title/Abstract])) OR (bone remodeling[Title/Abstract])) OR (osteocalcin[Title/Abstract])) OR (osteoprotegerin[Title/Abstract])) OR (alkaline phosphatase[Title/Abstract])) OR (procollagen type I N-terminal peptide[Title/Abstract])) OR (((((((((bone resorption[MeSH Terms]) OR (collagen type I trimeric cross-linked peptide[MeSH Terms])) OR (tartrate resistant acid phosphatase[MeSH Terms])) OR (bone resorption[Title/Abstract])) OR (collagen type I trimeric cross-linked peptide[Title/Abstract])) OR (C-terminal telopeptide[Title/Abstract])) OR (N-terminal telopeptide[Title/Abstract])) OR (deoxypyridinoline[Title/Abstract])) OR (tartrate resistant acid phosphatase[Title/Abstract])))) AND (humans NOT animals)**

1141

EMBASE

History

#13 #11 AND #12 207

#12 humans NOT animals 469,106

#11 #3 AND #10 5,110

#10 #6 OR #9 423,553

#9 #7 OR #8 100,141

#8 'bone resorption':ab,ti OR 'collagen type i trimeric cross-linked peptide':ab,ti OR 'c-terminal telopeptide':ab,ti OR 'n-terminal telopeptide':ab,ti OR 'deoxypyridinoline':ab,ti OR 'tartrate resistant acid phosphatase':ab,ti

43,502

#7 'bone resorption'/exp OR 'collagen type i trimeric cross-linked peptide' OR 'tartrate resistant acid phosphatase'/exp

86,325

#6 #4 OR #5 365,773

#5 'ossification':ab,ti OR 'bone metabolism':ab,ti OR 'bone formation':ab,ti OR 'osteogenesis':ab,ti OR 'bone turnover':ab,ti OR 'bone remodeling':ab,ti OR 'osteocalcin':ab,ti OR 'osteoprotegerin':ab,ti OR 'alkaline phosphatase':ab,ti OR 'procollagen type i n-terminal peptide':ab,ti

232,900

#4 'bone remodeling'/exp OR 'osteogenesis'/exp OR 'osteocalcin'/exp OR 'osteoprotegerin'/exp OR 'alkaline phosphatase'/exp OR 'procollagen type i n-terminal peptide'

288,748

#3 #1 OR #2 362,529

#2 'non insulin dependent diabetes mellitus'/exp

313,673

#1 'diabetes mellitus, type 2':ab,ti OR 'type 2 diabetes mellitus':ab,ti OR 'type 2 diabetes':ab,ti OR 'type 2 diabetic':ab,ti OR t2dm:ab,ti OR t2d:ab,ti

255,816


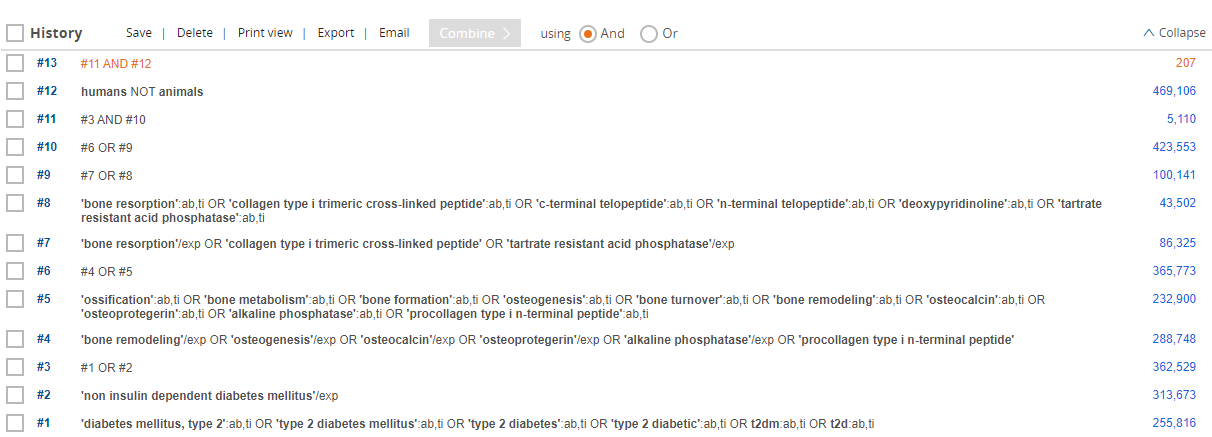


EBSCOhost

Search ID#

S7

S5 AND S6

Results (71)

S6

humans NOT animals

Results (3,109,826)

S5

S1 AND S4

Results (258)

S4

S2 OR S3

Results (25,888)

S3

SU ( bone resorption OR collagen type I trimeric cross-linked peptide OR tartrate resistant acid phosphatase ) OR AB ( bone resorption OR collagen type I trimeric cross-linked peptide OR C-terminal telopeptide OR N-terminal telopeptide OR deoxypyridinoline OR tartrate resistant acid phosphatase )

Results (4,671)

S2

SU ( bone remodeling OR osteogenesis OR osteocalcin OR osteoprotegerin OR alkaline phosphatase OR procollagen type I N-terminal peptide ) OR AB ( ossification OR bone metabolism OR bone formation OR osteogenesis OR bone turnover OR bone remodeling OR osteocalcin OR osteoprotegerin OR alkaline phosphatase OR procollagen type I N-terminal peptide )

Results (23,743)

S1

SU diabetes mellitus, type 2 OR AB ( Diabetes mellitus, type 2 OR Type 2 diabetes mellitus OR Type 2 diabetes OR Type 2 diabetic OR T2DM OR T2D )

Results (40,568)


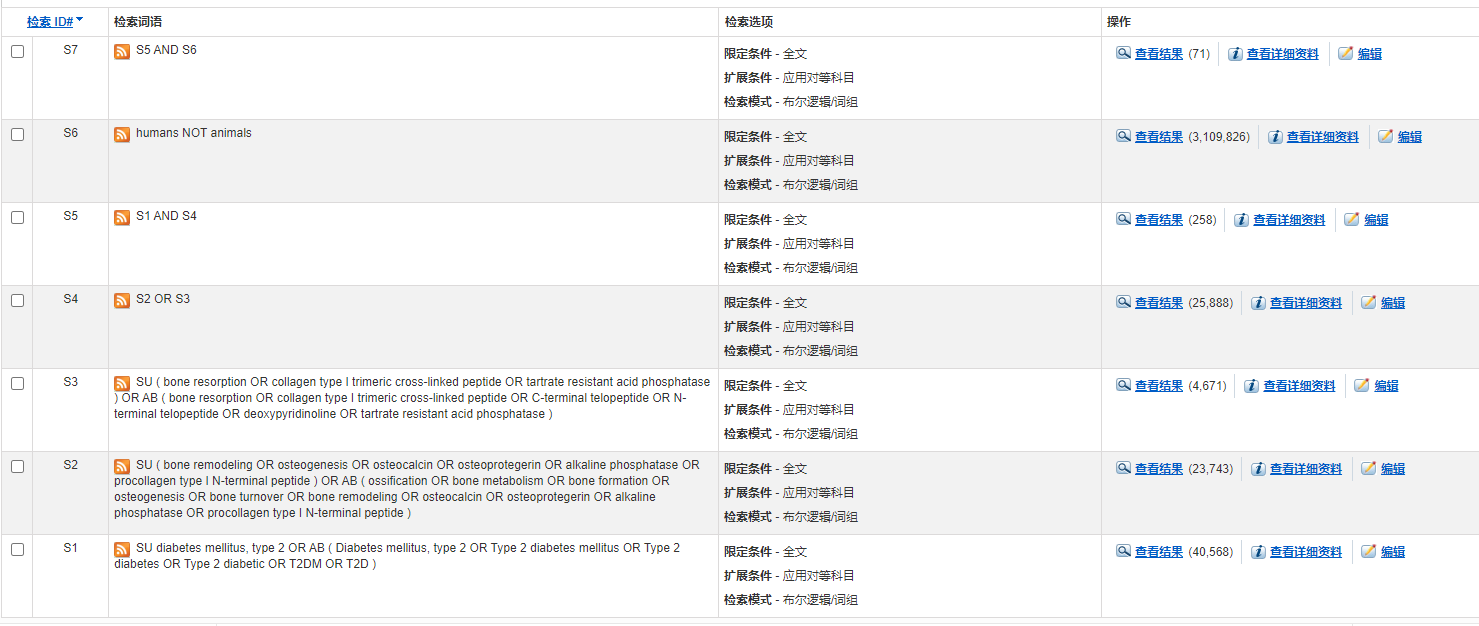


Cochrane library

Search help View/Share saved searches Save this search

Print search historyView fewer lines

#1

MeSH descriptor: [Diabetes Mellitus, Type 2] explode all trees

MeSH

20291

#2

(Diabetes mellitus, type 2 OR Type 2 diabetes mellitus OR Type 2 diabetes OR Type 2 diabetic OR T2DM OR T2D):ti,ab,kw (Word variations have been searched)

(Word variations have been searched)S Limits

55951

#3

#1 or #2

Limits

55952

#4

MeSH descriptor: [Bone Remodeling] explode all trees

MeSH

2751

#5

MeSH descriptor: [Osteogenesis] explode all trees

MeSH

367

#6

MeSH descriptor: [Alkaline Phosphatase] explode all trees

MeSH

1286

#7

MeSH descriptor: [Osteocalcin] explode all trees

MeSH

740

#8

MeSH descriptor: [Osteoprotegerin] explode all trees

MeSH

135

#9

#4 or #5 or #6 or #7 or #8

Limits

4399

#10

(ossification OR bone metabolism OR bone formation OR osteogenesis OR bone turnover OR bone remodeling OR osteocalcin OR osteoprotegerin OR alkaline phosphatase OR procollagen type I N-terminal peptide):ti,ab,kw (Word variations have been searched)

(Word variations have been searched)S Limits

19321

#11

#9 or #10

Limits

20319

#12

MeSH descriptor: [Bone Resorption] explode all trees

MeSH

2418

#13

MeSH descriptor: [Tartrate-Resistant Acid Phosphatase] explode all trees

MeSH

58

#14

#12 or #13

Limits

2459

#15

(bone resorption OR collagen type I trimeric cross-linked peptide OR C-terminal telopeptide OR N-terminal telopeptide OR deoxypyridinoline OR tartrate resistant acid phosphatase):ti,ab,kw (Word variations have been searched)

(Word variations have been searched)S Limits

4969

#16

#14 or #15

Limits

6235

#17

#11 or #16

Limits

22227

#18

#3 AND #17

Limits

754

#19

humans NOT animals

Limits

650294

#20

#18 AND #19

Limits

221


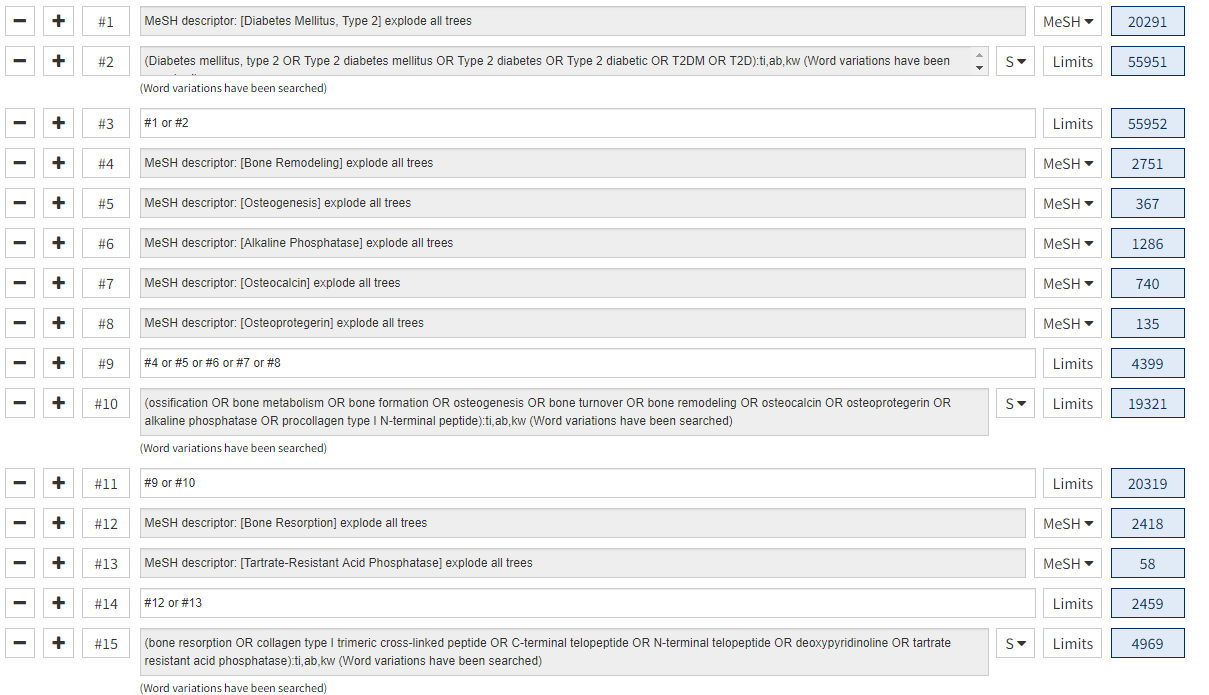


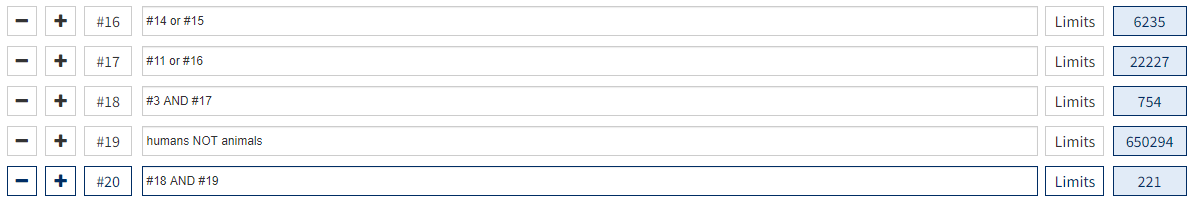


Web of science


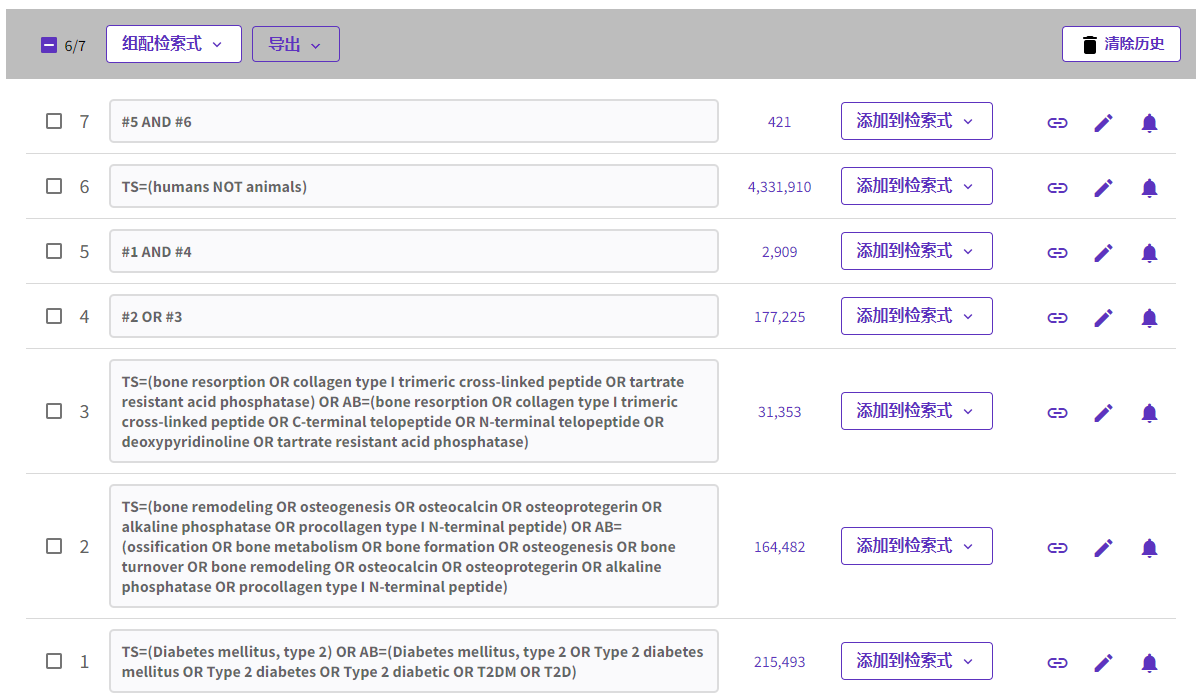


TS=(Diabetes mellitus, type 2) OR AB=(Diabetes mellitus, type 2 OR Type 2 diabetes mellitus OR Type 2 diabetes OR Type 2 diabetic OR T2DM OR T2D)

TS=(bone remodeling OR osteogenesis OR osteocalcin OR osteoprotegerin OR alkaline phosphatase OR procollagen type I N-terminal peptide) OR AB=(ossification OR bone metabolism OR bone formation OR osteogenesis OR bone turnover OR bone remodeling OR osteocalcin OR osteoprotegerin OR alkaline phosphatase OR procollagen type I N-terminal peptide)

TS=(bone resorption OR collagen type I trimeric cross-linked peptide OR tartrate resistant acid phosphatase) OR AB=(bone resorption OR collagen type I trimeric cross-linked peptide OR C-terminal telopeptide OR N-terminal telopeptide OR deoxypyridinoline OR tartrate resistant acid phosphatase)
